# Full Thickness Retinal Hole Formation after Nd:YAG Laser Hyaloidotomy in a Case with Valsalva Retinopathy

**DOI:** 10.1155/2018/2874908

**Published:** 2018-06-03

**Authors:** Yasin Sakir Goker, Kemal Tekin, Cemile Ucgul Atilgan, Pinar Kosekahya, Pelin Yilmazbas

**Affiliations:** ^1^Department of Ophthalmology, University of Health Sciences, Ulucanlar Eye Training and Research Hospital, Ankara, Turkey; ^2^Kars State Hospital, Ophthalmology Department, Kars, Turkey

## Abstract

A 27-year-old male was presented with a sudden onset of visual loss in his right eye. A secondary care center referred the patient with fundus photographs which were screened 4 days before and after the Nd: YAG laser hyaloidotomy treatment. Snellen acuity was 10/10 in both eyes. Fundus examinations revealed a retinal pigment epithelium (RPE) alteration at the margin of the inferior temporal arterial vascular arcade in the right eye and resolved preretinal and subretinal hemorrhages were seen in the macula. A diagnosis of Valsalva retinopathy was made based on the history and the treatment photographs of Nd:YAG laser hyaloidotomy. At 1^st^ month examination all hemorrhages were resolved but RPE alterations were still at the margin of the inferior temporal arterial vascular arcade. The optical coherence tomography angiography (OCTA) images revealed 2 lesions. On en face OCT angiogram of OCTA full thickness retinal hole formation and ellipsoid zone damage at the superior and inferior margin of the inferior temporal arterial vascular arcade were seen. Superficial vascular plexus was also damaged at that region. The projection of the evacuation of blood from subhyaloid space and the full thickness retinal hole formation were the same, indicating that the partial and full thickness retinal holes were created by the laser treatment.

## 1. Introduction

Valsalva retinopathy was firstly reported by Thomas Duane as ‘a particular form of retinopathy, preretinal, and hemorrhagic in nature, secondary to a sudden increase in intrathoracic pressure' in 1972 [1]. The name Valsalva comes after the Italian anatomist Antonio Maria Valsalva who defined the Valsalva ligaments. The Valsalva maneuver comprises forcible exhalation against a closed glottis. It is frequently unilateral but may be bilateral condition that occurs when increased intrathoracic or intra-abdominal pressure transmitted to the eye. A sharp rise in the intraocular venous pressure causes the rupture of superficial perifoveal retinal capillaries spontaneously. Visual prognosis depends on the size of the vessel involved and the location of the hemorrhage; subretinal, intraretinal, and/or subhyaloid [2]. The hemorrhage resolves spontaneously in most of the cases over a span of several weeks. Large preretinal hemorrhages have been treated with Nd:YAG laser to facilitate evacuation of the blood into the vitreous and rapid resolution [3]. On the other hand Nd:YAG laser treatment has complications including macular hole, retinal detachment, and epiretinal membrane formation [4–8]. Herein we present a rare complication: partial and full thickness retinal hole formation according to Nd:YAG laser hyaloidotomy in a case with Valsalva retinopathy.

## 2. Case Report

**Figure 1:**
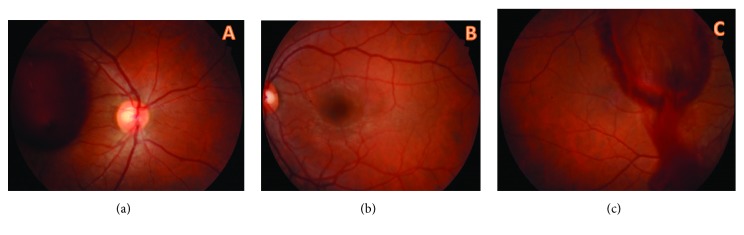
Fundus photographs which were screened in the second care clinic. (a) Premacular hemorrhage was seen in the right eye of the patient. (b) Normal macula of the left eye. (c) After the Nd:YAG laser hyaloidotomy treatment, evacuation of blood from subhyaloid space was seen in the right eye.

A 27-year-old male was referred to our hospital by a secondary care center due to a sudden onset of visual loss in his right eye, which occurred while weight-lifting in sports gym 4 days previously. The clinic referred the patient with fundus photographs which were screened 4 days before (Figure 1) and after the Nd: YAG laser hyaloidotomy treatment. Snellen acuity was 10/10 in both eyes. There was no afferent papillary defect and anterior segment examination was normal. The intraocular pressures were measured as 14 mmHg in both eyes.

**Figure 2:**
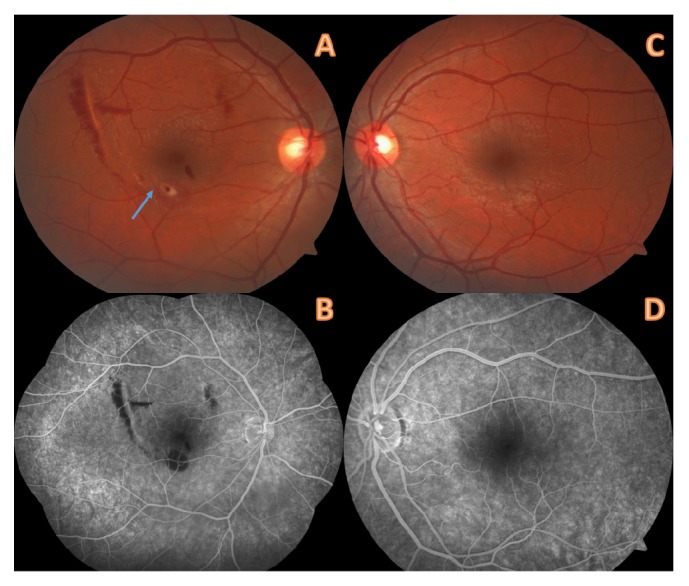
First day of examination of the patient in our clinic. (A) A retinal pigment epithelium (RPE) alteration was seen at the margin of the inferior temporal arterial vascular arcade in the right eye (blue arrow) and resolved preretinal and subretinal hemorrhages were also seen. (B) Fundus fluorescein angiography of the same eye. (C-D) Normal macula of the left eye.

Dilated fundus examinations revealed a normal macula in left eye. But there was a retinal pigment epithelium (RPE) alteration at the margin of the inferior temporal arterial vascular arcade in the right eye and resolved preretinal and subretinal hemorrhages were seen in the macula (Figure 2). A diagnosis of Valsalva retinopathy was made based on the history and the treatment photographs of Nd:YAG laser hyaloidotomy (Figure 1). The patient was also screened with fundus fluorescein angiography for any other vascular pathologies (Figure 2).

**Figure 3:**
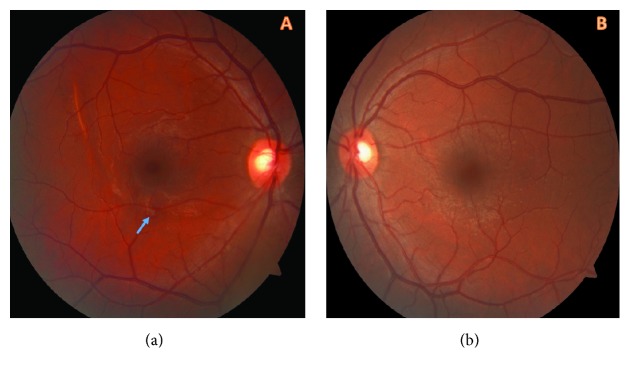
First month of examination of the patient. (a) All hemorrhages were resolved but RPE alterations were still seen at the margin of the inferior temporal arterial vascular arcade in the right eye (blue arrow). (b) Normal macula of the left eye.

**Figure 4:**
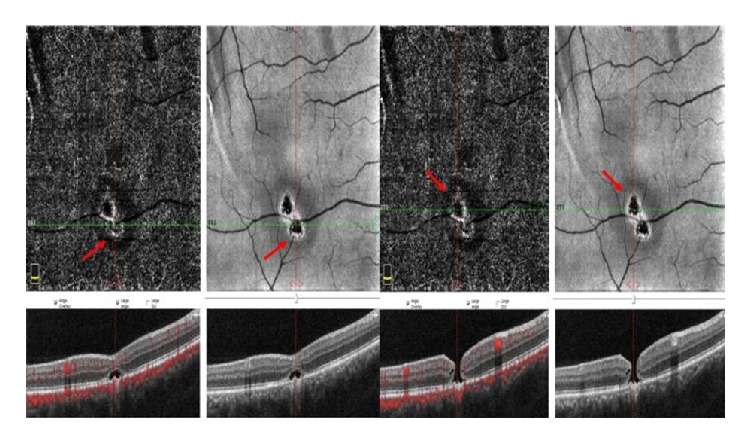
At first month of examination full thickness retinal hole formation and ellipsoid zone damage at the superior and inferior margin of the inferior temporal arterial vascular arcade were seen on en face optical coherence tomography (OCT) angiogram of OCT angiography (red arrows).

**Figure 5:**
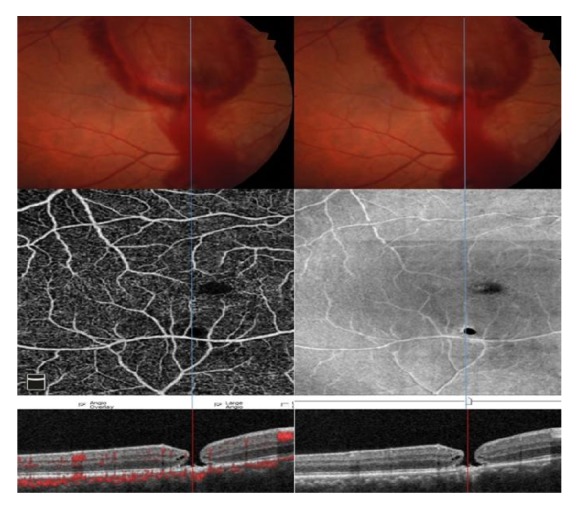
The projection of the evacuation of blood from subhyaloid space and the full thickness retinal hole formation were same (blue line). Superficial vascular plexus was damaged at that region.

At 1^st^ month of examination all hemorrhages were resolved but RPE alterations were still at the margin of the inferior temporal arterial vascular arcade in the right eye (Figure 3). The patient was screened with (OCTA) (OCTA; Avanti, Optovue RTVue XR). The OCTA images revealed 2 lesions. On en face OCT angiogram of OCTA full thickness retinal hole formation and ellipsoid zone damage at the superior and inferior margin of the inferior temporal arterial vascular arcade were seen (Figure 4). Superficial vascular plexus was also damaged at that region. The projection of the evacuation of blood from subhyaloid space and the full thickness retinal hole formation were same (Figure 5).

## 3. Discussion

Valsalva retinopathy can be treated by observation, Nd:YAG laser, and vitrectomy [3, 9–11]. Treatment choice depends on the duration, location, and the amount of the hemorrhage [12]. It is a self-limited event, and in most cases, the hemorrhage resolves within a month without any complications. The primary potential complication of wait and watch management is prolonged exposure of the retina to hemoglobin and iron. It can cause irreversible retinal damage and visual loss.

Faulborn et al. were firstly described Nd:YAG laser membranotomy in 1988 [13]. After that Gabel et al. reported Nd:YAG laser photodisruption of hemorrhagic detachment of the internal limiting membrane in 1989 and it has been used for Valsalva retinopathy since that report [3]. The effectiveness of Nd:YAG laser hyaloidotomy has been proven in most reports of Valsalva retinopathy patients [14]. Also Nd:YAG laser complications include macular hole, retinal detachment, and epiretinal membrane formation [4–8].

Nd:YAG laser was manufactured for anterior segment and commonly used for capsulotomy for pseudophakic eyes in posterior capsule opacification and iridotomy in angle-closure glaucoma. There have been many case reports and series in the literature describing Nd:YAG laser hyaloidotomy with different energy levels ranging from 2.5 to 50 mJ [15–19]. They reported relatively good success but the optimum energy level is not clear because of the density of the premacular hemorrhage and the broad range of the energy levels. Kuruvilla et al. started with 1.7 mJ laser power, titrated through 2.9 mJ, and achieved the appreciable effect at 3.8 mJ [15]. Gabel et al. reported energy levels up to 50 mJ were used with no retinal injury [3]. They postulated that the preretinal blood is thought to provide a buffer that protects the underlying retina from the laser energy. However Nd:YAG laser therapy has broken down mediated effect meaning that low pulse energies, below the break down threshold, may cause inadvertent retinal damage by its photodisruptive effect. Therefore it is critical to determine the optimum energy level for effective therapy.

In conclusion, Nd:YAG therapy can only be considered in premacular hemorrhages of at least 3 disc diameters in size. For an effective and safe laser treatment, three important criteria were crucial: location, energy level, and the choice of the lens. First is to choose the most appropriate position to facilitate evacuation of the blood into the vitreous at the inferior aspect of the fluid pocket. Secondly we suggest beginning with 1.5-2.0 mJ energy level with clear media titrating upwards gradually with 0.5 mJ intervals as required. Media opacities such as cataract, posterior capsular opacity, and vitreous hemorrhage will significantly modify the required power settings. And the third is to choose the proper contact macular lens. We use the Area Centralis (Volk Optical, Inc.; Mentor, OH) in our clinic. In our case we thought that inappropriate power settings were chosen and inadvertent retinal damage occurred by the photodisruptive effect of the Nd:YAG laser.
